# Weekly Variation of Rotavirus A Concentrations in Sewage and Oysters in Japan, 2014–2016

**DOI:** 10.3390/pathogens8030089

**Published:** 2019-06-26

**Authors:** Erika Ito, Jian Pu, Takayuki Miura, Shinobu Kazama, Masateru Nishiyama, Hiroaki Ito, Yoshimitsu Konta, Gia Thanh Nguyen, Tatsuo Omura, Toru Watanabe

**Affiliations:** 1 The United Graduate School of Agricultural Sciences, Iwate University, Iwate 020-8550, Japan; 2 Faculty of Information Networking for Innovation and Design, Toyo University, Tokyo 115-0053, Japan; 3 Department of Environmental Health, National Institute of Public Health, Saitama 351-0197, Japan; 4 Department of Urban Engineering, The University of Tokyo, Tokyo 113-8656, Japan; 5 Department of Food, Life and Environmental Sciences, Yamagata University, Yamagata 997-8555, Japan; 6 Center for Water Cycle, Marine Environment and Disaster Management, Kumamoto University, Kumamoto 860-8555, Japan; 7 New Industry Creation Hatchery Center, Tohoku University, Miyagi 980-8579, Japan; 8 Department of Environmental and Occupational Health, College of Medicine and Pharmacy, Hue University, Hue city 530000, Vietnam

**Keywords:** rotavirus, oyster, sewage, real-time PCR

## Abstract

Concentrations of rotavirus A, in sewage and oysters collected weekly from September 2014 to April 2016 in Japan, were investigated using RT-qPCR; results showed up to 6.5 log_10_ copies/mL and 4.3 log_10_ copies/g of digestive tissue (DT) in sewage and oysters, respectively. No correlation was found between rotavirus concentration in sewage and oysters and cases of rotavirus-associated gastroenteritis.

## 1. Introduction

Rotavirus is the major cause of acute gastroenteritis that leads to deaths in infants and young children worldwide. Before vaccines were introduced, rotavirus caused 20–40 deaths annually in the U.S. alone, and mortality was much higher in sub-Saharan Africa and South Asia [1,2]. Moreover, rotavirus was associated with up to 88% of all hospital-associated diarrheal episodes in Japan, before the introduction of vaccines, and led to 2–18 deaths every year [3,4]. While rotavirus can infect all age groups, young groups are mainly affected. Among 4072 rotavirus-associated gastroenteritis cases during the period of 2005−2010 in Japan, approximately 75% were 0- to 2-year-old babies [5]. Various vaccines have been licensed worldwide, including Rotarix, RotaTeq, Rotavac, and Rotasiil [6]. The first two have been commercially available in Japan since November 2011 and July 2012, respectively, for voluntary vaccination. Previous research has shown a decline of rotavirus deaths in 2013, after entering the vaccine era, but mortality in children <5 years remained high globally (197,000−233,000 deaths estimated) [7]. While norovirus has been well recognized to contaminate oysters, causing high levels of gastroenteritis in temperate regions during winter months, rotavirus was also detected in 0.3% to 16.7% of cases with oyster-associated gastroenteritis [8,9]. Although rotavirus has been detected in farmed oysters at rates of 3.3%–44.4% [9,10,11], information about their level of contamination in the environment and its seasonal variation remains limited. In this study, we performed long-term weekly monitoring of oysters at a cultivation site in Japan, tracking changes in viral loads across different seasons. The incidence of rotavirus in sewage in the same area was also simultaneously monitored, since it is likely to be the main source of rotavirus content in the oysters.

## 2. Results and Discussion

**Figure 1:**
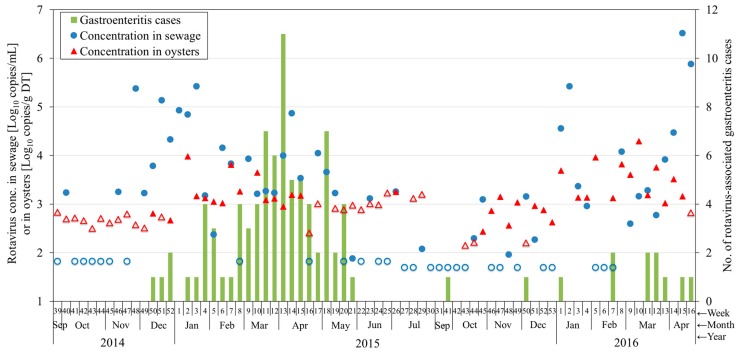
Rotavirus A concentration in sewage and oyster samples together with the number of rotavirus-associated gastroenteritis cases (green columns) in Miyagi, Japan. Empty circles and empty triangles represent half of the detection limit (LOD) in sewage and oysters, respectively, where rotavirus may exist, but below the detection limit. The weeks in which no oyster sample was collected or was tested positive due to low murine norovirus (MNV) recovery rate are considered invalid and left blank; The corresponding number of genomes for quantification cycles (C_q_ values) of 40 varied across qPCR runs, and the weight of digestive tissue was different in each oyster sample. Thus, LOD for each sewage and oyster sample was different; half of LOD has been shown in the figure for convenience of presentation.

Data related to rotavirus A contamination in sewage and oyster samples, as well as to gastroenteritis cases, are presented in Figure 1. Among the samples collected between 24 September 2014 and 21 April 2016, the highest rotavirus concentration obtained from sewage and oyster samples was 6.5 log_10_ copies/mL and 4.3 log_10_ copies/g of digestive tissue (DT), respectively. Approximately 62.2% (46 of 74 weeks) of sewage and 57.8% (37 of 64 weeks) of oyster samples were positive for rotavirus, which is much higher than the positivity rates reported in previous studies. In Thailand, rotavirus was detected in 27.1% (16 of 59), 9.1% (5 of 55), and 5.4% (5 of 110) of river water, irrigation canal water, and cultured oyster samples, respectively [10]. A wide range of positivity rates for rotavirus has been reported in oysters from different regions. Approximately 3.3% (5 of 150) of farmed oysters in China were found to be contaminated with rotavirus [11], whereas a comparatively higher positivity rate (44.4%, 4 of 9) was found in oysters, related to an outbreak in Southern France [9]. However, we cannot deny the possibility that the positivity rate was influenced by differences in our detection methodologies.

Humans, who consume oysters grown in contaminated water, are at a risk of rotavirus infection. Rotavirus concentrations reached 2.3 log_10_ PFU/g DT in oysters cultured for 48 h in artificial seawater, containing 10^4^ PFU/mL of the rotavirus strain Wa [12]. In Japan, 1 of 286 fecal specimens was found to be positive for rotavirus in 88 oyster-associated gastroenteritis outbreaks [8]. Approximately 16.7% (2 of 12) of patients with shellfish-associated gastroenteritis shed rotaviruses, along with other viruses, such as astrovirus, Aichi virus, and enterovirus [9]. Our cross-correlation analysis found that log transformed norovirus GII concentrations in sewage and oysters was significantly correlated with the number of gastroenteritis cases in the same study area [13]; however, none of the cross-correlation coefficients in this study was statistically significant at the 95% confidence level. There are several possible explanations for this inconsistency. First, the number of rotavirus-associated gastroenteritis cases, reported each week, was small, ranging from 0 to 11, and 56.8% of the weeks (42 of 74) reported no patient with rotavirus-associated disease, according to the Infectious Diseases Weekly Report of Miyagi Prefecture [14]. Second, shedding of rotavirus from domestic animals could cause a high load of rotavirus in seawater and oysters, whereas only those shed by humans could be detected in sewage, since over 99% of animal wastes do not enter municipal sewage system in Japan [15]. On the other hand, infants that receive rotavirus vaccine can shed up to 10^7^ copies in one gram of stool [16]; rotavirus vaccine (rotarix)-derived strains were found in six stool samples from pediatric clinics in Japan [17]. Therefore, there is a chance that feces from 5- or 6-month-old vaccinated babies also enter sewage, adding to the complexity of rotaviruses shed from humans. Third, despite the high concentration of rotavirus in seawater, caused by its low removal efficiency by wastewater treatment processes compared to that of norovirus [18,19], different stabilities were observed for different viruses in seawater [20], and different accumulation efficiencies in oysters were observed for different virus strains [21]. This could explain the weak correlations observed in this study. Weekly variation of rotavirus concentrations in sewage and oysters provide new insights into the distribution of rotavirus in wastewater, marine water, and shellfish. 

## 3. Materials and Methods 

Municipal sewage (1 L) and oyster (9 in number) samples were collected weekly (73 weeks in total) from Miyagi Prefecture, Japan, between 24 September 2014 and 21 April 2016. Virus particles were concentrated from sewage samples by polyethylene glycol precipitation [22]. Digestive tissue (DT) of each individual oyster was excised, and the virus extracted following a previously described protocol [23]. Approximately 1.5 mL viral supernatant was generated from each oyster. Three supernatants were pooled to form one oyster composite, and 3 oyster composites from each week were used for RNA extraction. Viral RNA was extracted from sewage and oyster samples as described earlier [23]. Complementary DNA (cDNA) was generated via reverse transcription using the iScript Advanced cDNA Synthesis Kit (Bio-Rad, Hercules, CA, USA) and a T100 thermal cycler (Bio-Rad), following the manufacturer’s instructions. Rotavirus A was quantified from the cDNAs by quantitative real-time PCR (qPCR) targeting rotavirus on a CFX96 Touch Real-Time PCR Detection System (Bio-Rad), using previously developed primers and probes [24]. Murine norovirus (MNV) was added to samples during the viral extraction step as a whole-process control [22]. Samples with MNV recovery rates higher than 1% were considered valid [25]. Quantification by qPCR was performed in accordance with the minimum information for the publication of real-time quantitative PCR experiments (MIQE) guidelines [26], and samples with quantification cycles (C_q_ values) below 40 were considered positive for rotavirus.

Lag time (±7 weeks) was studied between log-transformed rotavirus concentrations in sewage and oyster samples (collected weekly) and the number of rotavirus-associated gastroenteritis cases reported weekly by 5 pediatric sentinel clinics in Miyagi Prefecture [15], using cross-correlation analysis [27]. A time-series cross-correlation coefficient of ±7 weeks was calculated to identify correlation between the following events: (1) Occurrence of gastroenteritis cases, (2) shedding of viruses from infected individuals into sewage, and (3) contamination of oysters with viruses. In samples where rotavirus was not detected positively, the incidence of rotavirus was estimated to be half of the limit of detection (LOD) to permit cross-correlation analysis [28,29].
